# Comparison of application values of CT and MRI in the diagnosis of early Lacunar Infarction

**DOI:** 10.12669/pjms.341.13619

**Published:** 2018

**Authors:** Yuqing He, Liping Wei, Wenbo Li

**Affiliations:** 1 Yuqing He, Department of Neurology, Ward-I, Luoyang Central Hospital, Affiliated to Zhengzhou University, Zhengzhou, 471000, China; 2 Liping Wei, Department of Neurology, Ward-I, Luoyang Central Hospital, Affiliated to Zhengzhou University, Zhengzhou, 471000, China; 3 Wenbo Li, Department of Neurology, Ward-I, Luoyang Central Hospital, Affiliated to Zhengzhou University, Zhengzhou, 471000, China

**Keywords:** Computed tomography, Lacunar infarction, Magnetic resonance imaging

## Abstract

**Objective:**

To analyze and compare the values of computed tomography (CT) and magnetic resonance imaging (MRI) in the diagnosis of early lacunar infarction.

**Methods:**

Eighty-eight patients with early lacunar infarction who were admitted to the hospital were selected as research subjects, and all of them underwent MRI and CT. The study was conducted at our hospital between May 2015 to July 2016.

**Results:**

Four hundred and forty-one lesions were detected by MRI, and 145 were detected by CT. Most of the lesions were located at the thalamus, frontal lobe and parietal lobe. The detection rate of small lesions with a diameter less than 5 cm using MRI was significantly higher than that with CT, and the difference had statistical significance (P<0.05). Forty-nine cases who reached hospital within six hours were scanned in which no images were observed by CT, while small flake-like or spotted images were observed in 47 cases by MRI. The efficacy of MRI in displaying early lesions and micro lesions was superior to that of CT.

**Conclusion:**

In the diagnosis of early lacunar infarction, the detection rate of lesions with MRI is significantly higher than that with CT. MRI can clearly display the specific conditions of lesions, which is worth clinical promotion.

## INTRODUCTION

Lacunar infarction is a common cerebrovascular disease in clinics.^1^,^2^ After the infarction of central branch of cerebral artery is induced by arteriolosclerosis, cerebral tissues whose blood is supplied by the central branch induce softening lesions, leading to the formation of cavity. The common pathogenesis for lacunar infarction includes hypertension, diabetes, arteriosclerosis, etc. With the enhancement of living standard, the incidence of the above diseases has become increasingly higher and the incidence of lacunar infarction have also occurred. Lacunar infarction threatens the health and living quality of human beings. But as the lesions of lacunar infarction are small, there are no obvious clinical manifestations. Therefore, it is essential to find out methods to determine the position of early lacunar infarction and the size of occlusive blood vessels.

With the constant update of equipment and the advancement of technologies, magnetic resonance imaging (MRI) and computed tomography (CT) which develops rapidly are the frequently used imaging diagnostic method at present. MRI and CT with different technical characteristics have different efficacy in diagnosing different diseases.^3^ Luo XH et al. considered that CT was suitable for diagnosing lesions with large volume and could evaluate the involvement scope and metastasis of tumor.^4^ MRI has important values in determining whether lesions are benign or malignant. The detection rate of necrotic lesions with MRI was higher than that with CT; moreover MRI can precisely position lesions and determine the scope of lesions. Enas A et al. found that the diagnostic efficacy of MRI was superior to that of CT in the diagnosis of acute cerebral infarction.^5^ A clinical practice found that MRI had certain deficiencies such as small spatial resolution and inability to examine patients who were installed with cardiac pacemaker.^6^ CT can clearly display lesions based on the density changes of lesion tissues. If there was no apparent necrosis and cystic changes in lesions and moreover tissues have small density changes, they will not be detected by CT. But few studies comparing the efficacy of MRI and CT in the diagnosis of early lacunar infarction are available. To further analyze the values of MRI and CT in diagnosing lacunar infarction, this study examined and diagnosed 88 patients with early lacunar infarction using MRI and CT.

## METHODS

Eighty-eight patients with early lacunar infarction who were admitted to the hospital between May 2015 and July 2016 were selected. All the patients were diagnosed according to the diagnostic criteria of early lacunar infarction^7^; all of them underwent CT and MRI. The age was between 39 and 78 years (average 63.23±6.75 years). There were 42 males and 46 females. There were 27 cases of self-reported sensory disturbance, eight cases of emesis, 19 cases of speech disorder, 42 cases of facial paralysis, 40 cases of drowsiness and five cases of headache and dizziness. This study was approved by the ethics committee of our hospital, and all the patients signed informed consent.

### Inclusion and Excxlusion criteria

Patients who had malignant tumor, metal diseases, cardiac diseases or blood diseases, large-scale cerebral infarction and incomplete data were excluded. Patients who had complete data, were diagnosed as lacunar infarction, and developed no hematological system diseases, diseases associated to the heart, lung, liver and kidney and organic pathological changes were included.

A 16-layer spiral CT (Shenyang Dongruan Co. Ltd.; Neuviz, Dau I) (layer thickness: 5 mm; 0.5 T) was performed; the patients underwent conventional axial plain scan. As to MRI, MRI device (Ningbo Xingaoyi; OPER-0.5) which had layer thickness of 10 mm and applied 8-ring head coil was used to scan the brain. The scanning sequences were set as AX T1 weighed image (WI), T2WI, T2 fluid-attenuated inversion-recovery (FLAIR) and diffusion weighted imaging (DWI). The thickness of scanning layer and layer spacing was 10 mm; 18 layers were scanned. After examination, the examination results were identified and reviewed by more than two attending doctors. The number, size and morphology of the lesions were observed and analyzed.

### Statistical analysis

Data were statistically processed by SPSS ver. 21.0. Enumeration data were expressed as number of cases [N (%)], and comparison of categorical data between the two groups was performed using Chi-square test. Difference was considered as statistically significant if P<0.05.

## RESULTS

### Comparison of detection rates of lesions with CT and MRI

Four hundred and forty-one lesions were detected by MRI, while 145 lesions were detected by CT. If the detection rate of lesions using MRI was set as 100%, then the detection rate of lesions using CT was 32.9%. It suggested that the diagnostic efficacy of MRI was significantly better than that of CT, and the difference had statistical significance (X^2^=13.761, P<0.05). Most of the lesions were located at the thalamus, frontal lobe and parietal lobe. The lesions in different parts which were detected out by MRI were more than that by CT (Table-I).

**Table-I:** Detection results of the lesions in different parts [N (%)].

Examination method	MRI	CT
Brainstem	64(14.5)	20(13.8)
Cerebellum	61(13.8)	18(12.4)
Basal ganglia	57(12.9)	19(13.1)
Thalamus	72(16.3)	25(17.2)
Endocyst	52(11.8)	16(11.0)
Parietal lobe	68(15.4)	24(16.6)
Frontal lobe	67(15.2)	23(15.9)

Total	441(100)	145(100)

### Detection results of the size of lesions using MRI and CT

The comparison of the detection rates of lesions with a diameter larger than 5 mm and smaller than 5 mm suggested a statistically significant difference (P<0.05; Table-II).

**Table-II:** Detection results of the size of lesions [N (%)].

Examination methods	MRI	CT	X2	P
Number of lesions	441	145		
Diameter > 5 mm	131(29.7)	63(43.4)	7.968	<0.05
Diameter < 5 mm	310(70.3)	82(56.6)	9.164	<0.05

### The detection results of cases which were admitted within 6 hours using MRI and CT

Forty-nine cases which reached hospital within six hours were scanned. Images were not observed by CT, while 47 cases were observed having small flake-like or spotted images by MRI.

### Manifestations of CT and MRI

**Fig. 1:**
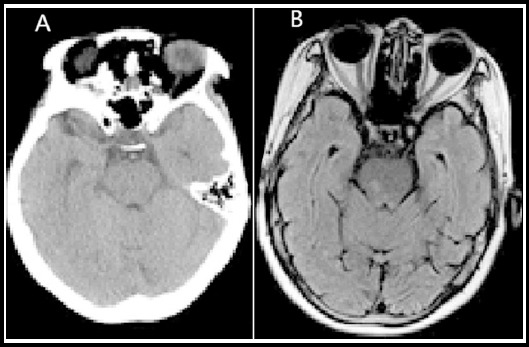
The lesions of patients with lacunar infarction in the examination with CT and MRI (A: Unclear display by CT; B: Clear display by MRI)

Spot-like, dotted and long circular lesions were observed by CT and MRI. In the images, the lesions were usually with unclear edge and without obvious occupying effect. As to density signals, there was a discrepancy between MRI and CT. In the examination with CT, lacunar infarction lesions were distributed in a low density; in the examination with MRI, isometric or longish T1 signals, long T2 signals and high T2FLAR and DWI signal were observed (Fig.1).

## DISCUSSION

Lacunar infarction which is featured by rapid onset and emergent disease condition can induce a series of complications if treatment is delayed. Therefore, effective treatment measures should be undertaken. However, treatment should be based on accurate diagnosis.^8^ Once lacunar infarction attacks, lesions with large volume are usually rare; metabolism of brain cells will be affected by ischemia and anoxia of cerebral tissues, which will restrain the functions of sodium and potassium ion pump, increase the concentration of sodium ions in lesions and induce cytotoxic edema of cerebral cells.^9^ A study suggested that lesions of lacunar infarction were usually small^10^, which was diagnosed as micro infarction and the maximum diameter of infarcted lesions was smaller than 15 mm.

Low density can be observed in the conventional CT images of the lesions of lacunar infarction. As the diagnostic time before and after onset has an obvious difference and the changes of lesions in cerebral tissues are small, it is difficult to find apparent improvement of abnormality in CT images, which can affect the formulation of early treatment strategies and may even result in wrong diagnosis. Relevant research results have demonstrated that CT is not sensitive to the changes of edema in lacunar infarction tissues, is poor in diagnosing cases whose onset is a short time before and is lowly sensitive to the diagnosis of lesions in the cerebellar hemispheres and posterior cranial fossa. MRI is highly sensitive to the distinction of pale gray and gray of infarction tissues and can clearly display the changes of interstitial edema.^11^ Some studies have demonstrated that lesions were usually displayed clearly 24 hours after onset. MRI with high resolution can display lesions earlier and is especially sensitive to T2WI.^12^-^15^ The results of this study also indicated that the detection rate of the lesions which occurred within six hours using MRI was significantly higher than that using CT and the number of the lesions discovered by MRI was larger than that by CT, which were consistent with the research results of Sun Z et al.^16^

Moreover in the examination of small lesions, it was found that the detection rates of the lesions using MRI was much higher than that using CT; in details, the number of infarcted lesions which was detected by MRI and had a diameter smaller than 5 mm was 310 (70.3%) and the number of infarcted lesions which was detected by CT and had a diameter smaller than 5 mm was 82 (56.6%), which were similar to the research results of Liu Feng.^17^ It indicated that MRI had a higher resolution. The cause for the above results might be the difference of density between the lesions with small diameter and surrounding tissues and the influence of body structure.^18^,^19^ The detection results of the brainstem and cerebellum using CT were poorer than that using MRI. MRI can more clearly display the pathological changes of special parts. Therefore the detection rate of micro infarction lesions using MRI was much higher than that using CT.

## CONCLUSION

The detection rate of early lacunar infarction using MRI is significantly higher than that using CT, and MRI performs well in displaying the morphology, size and location of lesions. Hence MRI is considered as the preferred choice for the diagnosis of early lacunar infarction and has a positive clinical diagnostic efficacy.

### Authors' Contribution

**YQH:** Study design, data collection and analysis.

**YQH, LPW & WBL:** Manuscript preparation, drafting and revising.

**YQH & WBL:** Review and final approval of manuscript.
